# Distraction during learning with hypermedia: difficult tasks help to keep task goals on track

**DOI:** 10.3389/fpsyg.2014.00268

**Published:** 2014-03-27

**Authors:** Katharina Scheiter, Peter Gerjets, Elke Heise

**Affiliations:** ^1^Multimedia Lab, Knowledge Media Research CenterTübingen, Germany; ^2^Hypermedia Lab, Knowledge Media Research CenterTübingen, Germany; ^3^Institute for Educational Psychology, Technische Universität BraunschweigBraunschweig, Germany

**Keywords:** hypermedia, distraction, volitional action control, goal conflicts, pending goal, seductive details

## Abstract

In educational hypermedia environments, students are often confronted with potential sources of distraction arising from additional information that, albeit interesting, is unrelated to their current task goal. The paper investigates the conditions under which distraction occurs and hampers performance. Based on theories of volitional action control it was hypothesized that interesting information, especially if related to a pending goal, would interfere with task performance only when working on easy, but not on difficult tasks. In Experiment 1, 66 students learned about probability theory using worked examples and solved corresponding test problems, whose task difficulty was manipulated. As a second factor, the presence of interesting information unrelated to the primary task was varied. Results showed that students solved more easy than difficult probability problems correctly. However, the presence of interesting, but task-irrelevant information did not interfere with performance. In Experiment 2, 68 students again engaged in example-based learning and problem solving in the presence of task-irrelevant information. Problem-solving difficulty was varied as a first factor. Additionally, the presence of a pending goal related to the task-irrelevant information was manipulated. As expected, problem-solving performance declined when a pending goal was present during working on easy problems, whereas no interference was observed for difficult problems. Moreover, the presence of a pending goal reduced the time on task-relevant information and increased the time on task-irrelevant information while working on easy tasks. However, as revealed by mediation analyses these changes in overt information processing behavior did not explain the decline in problem-solving performance. As an alternative explanation it is suggested that goal conflicts resulting from pending goals claim cognitive resources, which are then no longer available for learning and problem solving.

## Introduction

Recent years have seen rapid developments in the use of computer-based learning environments. Many of these environments are based on hypermedia structures, that is, network-like information structures where fragments of information such as text, pictures, or videos are stored in nodes that are interconnected by digital hyperlinks (Conklin, 1987). Hypermedia environments grant users control over their instruction in that they can select information units and choose the point of time as well as the pacing and sequence of presentation according to their goals (Dillon and Jobst, 2005; Scheiter and Gerjets, 2007; Scheiter, 2014). Accordingly, the same hypermedia environment may serve a multitude of different goals that guide information utilization, whereby each information unit can be classified as being more or less relevant with respect to a particular information goal. Accordingly, while pursuing a goal such as to learn about a specific topic or solve a particular problem students may encounter information in a hypermedia environment that is irrelevant to the primary learning or problem-solving goal.

In the current paper we were interested in the effects of information that is irrelevant to the current task, but nevertheless included in the hypermedia environment that is used to accomplish this task. This is a situation that has become omnipresent for many students, who nowadays are often using the Internet as their primary resource for finding information relevant to their educational goals (e.g., for doing their essay assignments, looking up answers to a specific question, etc.; cf. Lenhart et al., 2001). On the one hand, rapid access to a vast amount of information available through the Internet is seen as beneficial because it allows students to come up with a multifaceted mental representation of the content in question; on the other hand, it imposes high cognitive demands on users in terms of evaluating whether a specific piece of information is relevant to the task at hand and of keeping track of the current goal (Braasch et al., 2009). Recent research has shown that students largely vary in their ability to evaluate information relevance, which is predictive of their task performance: successful students have been shown to reflect upon the relevance of the information that they find while browsing the Internet and monitor how well a piece of information will help them to accomplish their educational objective, whereas less successful students appear to be less aware about information relevance when searching the Internet (Braasch et al., 2009; Goldman et al., 2012). The latter students' navigation behavior appears more erratic and less attuned toward the educational goal. This may be because these students are distracted by task-irrelevant, albeit potentially interesting information that they encounter.

Carmel et al. (1992) speak of general purpose browsing when information search is guided by interest. In this case, *transient search goals* are assumed to be formed during browsing that change over time depending on the local context provided in the hypermedia environment (i.e., context-sensitive browsing according to Hirashima et al. (1997). These transient browsing goals may compete with the current primary task for execution. Moreover, the information that is irrelevant to the primary task may even be relevant to other, currently pending tasks that a user is aware of when entering the hypermedia environment. Although there may be sufficient later opportunities to perform these pending tasks (i.e., after having accomplished the primary task), these pending tasks may nevertheless compete with the learning task for being executed (i.e., *pending goals*). Thus, even though students' navigation behavior may sometimes appear erratic, it is goal-driven, but these goals are different from the primary educational goal of finding task-relevant information that will help to solve a learning and problem-solving task.

In the present paper we were interested in the effects of transient as well as pending goals during learning and problem solving with a hypermedia environment. In particular, we studied how such goals would impact students' problem-solving performance as well as their information processing behavior in terms of retrieving and reading information that was either relevant or irrelevant to the primary task. Against the backdrop of theories of volitional action control it was investigated how the difficulty of the primary (learning and problem solving) task would moderate the effects of transient and pending goals, respectively.

### Transient search goals during learning and problem solving with hypermedia

Presumably everybody who has ever tried finding a specific information on the Internet has made the experience that one may easily get carried away and click on hyperlinks that will provide access to information unrelated to the primary search task. Such a retrieval of additional information is likely to be triggered by interest and curiosity evoked by the hyperlink's label. Carmel et al. (1992) or Hirashima et al. (1997) describe such a behavior as context-sensitive browsing, where a user's information access is guided by interest and transient search goals are formed. These goals are characterized by the fact that they may change rather quickly and be replaced by others depending on the information that is encountered and how well the information is suited to sustain interest. Thus, different from other goals that result from deliberate reasoning about expectancies and values associated with goal attainment, transient search goals are unlikely to have a lasting impact on behavior. Thus, it is not clear whether encountering interesting information and the transient goals that may arise from it will have a pronounced impact in the presence of an already existing learning or problem-solving goal.

Barab et al. (1996) assume that primary goals provide such a strong form of guidance that transient goals are unlikely to emerge. In particular, they assume that instructed or experimentally induced goals “constrain users' searches by removing external degrees of freedom (i.e., searches are not distracted by intention-irrelevant information on each screen)” (p. 387). In their study the experimental group had to choose a problem to be solved with information provided in a hypermedia system, whereas the control group had no specific goal when browsing that system. Comparing the two groups revealed differences in a number of strategic measures of information search and information utilization. Furthermore, higher standard deviations for these navigational measures in the no-goal group suggested that these students were guided by a variety of goals that were formed spontaneously during hypermedia navigation. Thus, whereas users in the no-goal condition seemed to be guided by transient browsing goals, users in the goal-condition focused on the accomplishment of the task that they had selected initially. Thus, according to the findings of Barab and colleagues the presence of potentially interesting, but task-irrelevant information does not appear to be sufficient to yield goals that are sufficiently strong to compete with the primary goal for execution.

On the other hand, research on the seductive details effect suggests that adding interesting, but task-irrelevant information to an instructional message (e.g., decorative illustrations, entertaining text messages) may have a negative effect on learning and problem solving (cf. Goetz and Sadoski, 1995; Harp and Mayer, 1998; Rey, 2012). Various explanations have been proposed for why seductive details hinder learning and problem solving (Harp and Mayer, 1998; Rey, 2012). In particular, seductive details may (a) distract students' attention away from processing task-relevant information toward task-irrelevant information (distraction), (b) trigger inappropriate schemas for encoding the information yielding an erroneous interpretation of it (diversion), (c) interrupt the construction of a coherent mental representation of the relevant information (disruption) or (d) demand cognitive resources which are then no longer available for the main task (depletion). Notably, only the first explanation should be visible in terms of students' information processing behavior, since according to this explanation distracted students should process relevant information less intensively when seductive details are present. Lehman et al. (2007) provided first evidence in line with this assumption: Their students spent less time reading relevant text when seductive details were present compared with when seductive details were absent. However, it is unclear from their study whether this change in information processing behavior was the cause of reduced performance or whether it just coincided with it. Nevertheless, it seems to be a plausible assumption that a less frequent retrieval of task-relevant pages should be associated with lower performance (see Naumann et al., 2007, for similar results during learning with hypermedia). Similarly, a more frequent retrieval of task-irrelevant information should yield reduced performance, especially if learners process this information at the expense of task-relevant information.

Based on the results on the seductive details effect, we assumed that the additional presentation of interesting, but task-irrelevant information in a learning and problem solving hypermedia environment would interfere with the primary problem-solving task. In particular, students were expected to show worse problem-solving performance and to process the interesting information at the expense of task-relevant information.

It is important to note however that our experimental setting differed from the settings in which the seductive details effect has been observed in how the irrelevant information was presented. In hypermedia environments, users commonly have to actively select the irrelevant information by clicking on a respective link, whereas the seductive details effect has been found when irrelevant information was presented on the same page as the relevant contents. As a consequence, negative effects of presenting interesting information in a hypermedia environment may be subtler than the ones usually found in seductive details research, where it is almost impossible to ignore the irrelevant information. On the other hand, providing interesting information in additional hypermedia nodes allowed us to register the time spent processing this information in a very parsimonious way by analyzing students' log files.

The impact of presenting interesting, but task-irrelevant information, which would presumably lead to the formation of transient browsing goals, was studied in Experiment 1 of the present paper. In Experiment 2, we studied how a pending goal would affect hypermedia-based learning and problem solving.

### Pending search goals during learning and problem solving with hypermedia

Pending goals in hypermedia-based learning and problem solving may arise if the user knows that s/he will have to perform a second task later on that is related to the information that is irrelevant to the primary task. That is, in contrast to transient goals pending goals are not spontaneously formed but already exist when a user starts working on a learning and problem-solving task. Pending goals may absorb cognitive resources needed for keeping them active until task pursuit and they may compete with the current learning and problem-solving goal for execution. There are findings from research on prospective memory demonstrating that pending goals reside in memory with a heightened state of activation (Goschke and Kuhl, 1993; Marsh et al., 1999). As a consequence of their specific status in memory, representations of prospective tasks will be activated very easily by related external cues (cf. Altmann and Trafton, 2002), which in turn may lead to interference, distraction, intrusion errors, and resource costs (cf. Li et al., 2000). Thus, when encountering information that is relevant to a pending task in a hypermedia environment, this information will be perceived as a good opportunity to engage in activities related to the accomplishment of the pending goal—even in situations where ample opportunity exists to postpone this task until the primary task has been completed.

Goal conflicts such as conflicts between pending goals and primary goals have been studied against the backdrop of theories on volitional action control (e.g., Heise et al., 1994b, 1997). In the context of learning and motivation, volitional action control refers to questions of how resources are allocated and managed during goal pursuit, how protective actions are taken toward goals, and how students cope with internal and external distractions (Corno and Kanfer, 1993). Volitional action control becomes especially important when “academic goals require sustained effort in the face of distractions and competing goals” (Corno and Kanfer, 1993, p. 305). One advantage of conceptualizing the present research against theories of volitional action control is that it allows to state more precisely the conditions under which pending goals (and possibly also transient goals) are most likely to have an impact on a primary task by causing distraction. In particular, theories of volitional action control suggest that goal competition will have less negative impact on a primary task, if the primary task is relatively difficult to accomplish.

### Task difficulty as a moderator

According to theories of volitional action control, an enhanced task difficulty of the primary goal leads to an increased level of effort that reduces distraction effects. In models of volitional action control this difficulty-related effort investment is interpreted as a volitional process that helps to maintain the current goal in the face of difficulties and to protect it against competing goals (cf. Kuhl, 1984; Gollwitzer, 1990; Corno, 1993; Heise et al., 1994a; Goschke, 2003). In line with this assumption it has been shown that distraction effects due to goal competition (as measured by impaired performance in a word-classification task) were stronger for easy tasks than for more difficult tasks (Heise et al., 1994b, 1997). Analogously, Czerwinski et al. (2000a,b) have demonstrated that instant messaging during computer-based information-search tasks resulted in performance impairments with respect to the search task, whereby these effects were moderated by the task relevance of the messages, the difficulty of the search task, and the search task's degree of completion at the time the distraction occurs.

Based on theories of volitional action control and the aforementioned findings we predicted that a pending goal related to interesting, but task-irrelevant information in a hypermedia environment would have a stronger negative impact on current task performance if the current task was easy rather than more difficult. Analogously, because a higher difficulty of the task should serve to prevent distraction, there should be less processing of task-irrelevant information at the expense of task-relevant processing when students work on more difficult tasks. Since we were interested in whether task difficulty would also moderate the effects of transient goals, the difficulty of the primary task was used as an independent variable in both experiments.

### Overview of experiments and hypotheses

In the present paper two experiments are reported which investigated how interesting, but task-irrelevant information would affect learning and problem solving in a hypermedia environment. In Experiment 1, interesting, task-irrelevant information was added under the assumption that students would form transient search goals regarding this information, which would then interfere with the primary goal of problem solving. In Experiment 2, students were also asked to carry out a search task regarding the interesting information, which they would have to work on after they had accomplished the primary problem-solving task. This instruction was assumed to lead to the formation of a pending goal lingering in memory while working on the problem-solving task.

According to Hypothesis 1, a transient goal as well as a pending goal related to interesting, but task-irrelevant information were assumed to lead to worse problem-solving performance than the presence of either no task-irrelevant information or less interesting information. Based on theories of volitional action control this effect was assumed to be visible only if the problem-solving task was rather easy, but not when it was more difficult.

According to Hypothesis 2, a transient goal as well as a pending goal were expected to lead to (more) time being spent on the processing of the task-irrelevant information (Hypothesis 2a) as well as to less time being spent on the processing of the task-relevant information (Hypothesis 2b).

According to Hypothesis 3, it was assumed that the negative effects of conflicting goals on problem-solving performance described in Hypothesis 1 would be mediated by the changes in information processing behavior described in Hypothesis 2.

## Experiment 1

This experiment aimed at testing whether giving learners the opportunity to retrieve interesting, but task-irrelevant information—thereby stimulating the formation of transient browsing goals—would adversely affect problem-solving performance. Negative effects of adding interesting, but task-irrelevant information were expected to be visible when working on an easy version of a problem-solving task, but not when working on a more difficult version. Moreover, it was expected that in the condition with an easy compared with a more difficult problem-solving task students would spend more time processing the interesting, task-irrelevant information, while at the same time processing task-relevant information to a lesser extent.

### Materials and methods

#### Participants and design

Sixty-six students (43 female, 23 male) of the University of Goettingen, Germany, participated in the experiment for either course credit or payment. Their average age was 24.94 years (*SD* = 3.95). Participation was voluntary. All participants had taken a course in introductory statistics and were therefore familiar with the domain chosen for experimentation, that is, probability theory. The study was based on a between-subjects 2 × 2-design with task difficulty (easy vs. difficult) and presence of task-irrelevant, but interesting information (with vs. without) as independent variables. Students were randomly assigned to one of the four experimental conditions with 17 participants serving in each of the two conditions containing additional interesting information and 16 participants serving in each of the two control conditions.

#### Materials

In the present studies we used a learning and problem-solving hypermedia environment on combinatorics called HyperComb. It conveys knowledge on how to calculate the number of possible arrangements or selections of elements as a prerequisite for determining the probability of complex events. The present version of HyperComb consisted of a short introduction to the domain of combinatorics where participants were instructed that they would have to solve three probability word problems. They were trained to use a multiple-choice form that they later needed for solving the test problems. The participants were further told that the worked-out examples, which would be used in order to convey information on different problem categories, would be available during the whole experiment (i.e., also during solving the test problems).

At the end of the introduction three word problems were presented on a single screen and one of them had to be selected to begin with. A navigation bar at the margin of the screen contained links to the worked examples as well as to the test problems and was accessible during the whole experiment. There was no formal distinction between a learning and a problem-solving phase in this environment. Rather, information necessary to solve the test problems could be retrieved during the whole course of the experiment. The test problems' difficulty depended on experimental condition (Table 1). In accordance with preliminary studies we manipulated their difficulty by using smaller numbers in the easy test problems and by stating them in a more familiar way than the difficult problems. Inspired by Ross and Kilbane (1997) in the easy test problems typical roles were assigned to the objects mentioned (e.g., knights choosing horses), whereas in the difficult problems reversed roles were assigned to the respective objects (e.g., horses choosing knights). The structural features of the test problems and thus their solution procedure were not affected by this manipulation of difficulty. Participants had to solve the problems by marking the correct problem category in a solution form, where the six problem categories were represented by their appropriate solution formulas. Additionally, participants had to specify the correct value for two variables out of a set of five alternatives, respectively.

**Table 1:** **Test problems**.

**Angler problem:** A club of vegetarian anglers has four (24) members. All vegetarian anglers have committed themselves to throw the fish they catch *directly* back into the lake (at the end of the day.). One day the club members, one after another, go fishing at a lake that is 8 (812) square meters in size and has five (220) fish in it: one (17) zander, one eel, one trout, one (200) pike(s) and one carp. *In the order of their age, all club members catch one fish*. (First, the eel bites into a hook. The angler throws the eel into a pail and continues fishing. Second, the trout catches the bait.) How do you calculate the probability of the oldest angler catching the eel and the second oldest catching the trout?
**Dog problem:** An animal home currently hosts 11 (81) dogs. 4 (14) of them are terriers, the remaining (67) are half-breeds. 2 (22) blond and 4 (14) brunette children come to the animal home wanting dogs as pets. To prevent the children from arguing over whom gets which dog, *the director asks the children to draw lots. First, the brunette children draw the lots, one each*. (the dogs are distributed by random. The name of each child is written on a dog biscuit, which are taken out of a bowl by the dogs. First, each of the terriers gets to choose one dog biscuit.) How do you calculate the probability of every brunette child getting a terrier?
**Knight problem:** 10 (110) knights participate in the 9th king's tournament. The king provides the tournament with 12 (122) horses. (that are able to talk by means of a magic potion. The horses start to pick the knights blindfold. The biggest horse gets to pick first, then the second biggest and so on.) *The knights have to pick their horses blindfold. The heaviest knight gets to pick first, then the second heaviest and so on*. How do you calculate the probability of the heaviest knight getting the biggest horse, the second heaviest knight getting the second biggest horse, and the third heaviest knight getting the third biggest horse?

*Text portions in italics were part of the problem statements for the easy versions only, whereas text portions in parentheses were used only in the difficult problems*.

Each of the six problem categories was illustrated by one worked-out example embedded in an interesting cover story related to issues of attractiveness and mate choice (see Table 2). Each worked-out example was presented on two separate pages. The first page contained the problem statement and a hyperlink referring to its solution. The solution page explained the structural features of the respective problem category, the appropriate solution formula, and its application to the example problem. Depending on experimental condition additional hyperlinks were embedded in each of the example problems that referred to interesting, but task-irrelevant information. The condition without additional interesting information (control condition) comprised only relevant information (i.e., the test problems and worked-out examples for the six problem categories) and consisted of 27 pages. In the condition with additional interesting information each worked-out example page was linked to one page with potentially interesting, but task-irrelevant information on attractiveness and mate choice. For instance, the top-200-list hyperlink in Table 2 referred to a list of billionaires, which was considered to be of personal interest to the learners. These pages that were directly linked to the worked-out examples were termed first-order irrelevant information pages. Additionally, we introduced second-order irrelevant information pages that could be retrieved by clicking hyperlinks embedded in first-order irrelevant information pages. Choosing first-order and second-order irrelevant information pages was interpreted as an active retrieval of irrelevant information. The condition with interesting information contained 18 additional first-order and second-order irrelevant pages (i.e., three irrelevant information pages for each worked-out example).

**Table 2:** **Worked-out example for the problem category “combination without replacement”**.

**Example problem:** Financial resources seem to be a key factor in mate choice, especially for women. Thus, it may be of interest that in the current top-200 list of the business magazine “Forbes” the 200 wealthiest people in the world are ranked by the sizes of their fortunes. What is the probability of selecting the 5 wealthiest persons out of this set of 200 people at random?
Please imagine this problem situation as well as possible and try to find a solution to the problem. When you have thought about the solution to this example problem please compare the solution that you have considered for this problem with this example solution.
**Example solution:** Combination problems are about the number of possibilities for selecting a subset of elements out of a set of elements without regard to the order in which they are selected (“*combinations*”). If no element can appear more than once in the selected subset, the problem is of the type combination without replacement.
The number *A* of possible combinations without replacement can be calculated by using the following formula:
*A* = *n*!/(*n* − *k*)!*k*!
*n* is the number of elements in a set that can be selected, *k* is the subset of selected elements and n! = n*(*n* − 1)*(*n* − 2)…*1.
The given example is about a selection out of a set of 200 persons (the top-200 list). This is the set of elements for selection (*n* = 200). The question asks the probability of randomly selecting the 5 richest persons out of this list, whereby the order of selecting the 5 persons is irrelevant. Therefore, the number of selected persons equals *k* = 5.
Inserting these values into the formula for combination without replacement, that is *A* = *n*!/(*n* − *k*)!*k*!, yields 200! / (200-5)! 5! = 2,535,650,040 combinations.
Thus the probability for one of these combinations (selecting the 5 wealthiest persons) equals 1/2,535,650,040 = 0.000000039%.

*Hyperlinks are underlined. The example problem and its solution were presented on separated pages*.

#### Dependent variables

As dependent measures we registered students' problem-solving performance, time on relevant information pages (i.e., worked-out examples and test problems) as well as time on actively retrieved irrelevant information in the condition with interesting information. For each of the three word problems the participants had to mark the correct problem category and values for the two variables in a multiple-choice form. One point was assigned for each correct answer so that a maximum of nine points was possible. The sum across all three problems was transformed into a percentage for easier interpretation. The time on relevant information pages as well as on task-irrelevant pages were recorded in seconds based on the log file data.

#### Procedure

Students were tested individually. After a short introduction to HyperComb, students entered the learning and problem-solving section of the environment. They were told that they could work through it at their own pace and go back and forth between information pages as they wished. There were no time limitations for the experiment. However, participants were told to work as quickly and as correctly as possible. Participants were told that in principle all information available might be helpful for solving the word problems. A single session lasted about 60 min.

#### Data analyses

In order to test the interaction hypotheses for problem-solving performance and time on relevant information we used regression analyses along with effect coding (cf. Abelson and Prentice, 1997; Niedenthal et al., 2002) since this captured our directional hypotheses most adequately. The basic idea behind contrast coding is to test whether a specific model (“focal contrast”), which is based on the hypothesized relative group differences, better fits the observed data than a number of independent (i.e., orthogonal), alternative models (“residual contrasts”). These residual contrasts are not necessarily meaningful in the sense that they represent alternative theoretical assumptions, but are defined according to formal requirements. If the focal contrast fits the data to a significant degree while the residual contrasts do not, it can be concluded that the hypothesized pattern of group differences describes the observed data accurately. If the focal contrast does not fit the data while the residual contrasts do, then the data do not conform to the hypotheses and are better explained by other models. If both the focal contrasts and the residual contrasts fit the model significantly, then the hypothesized group differences can be found in the data but the data are additionally explained by other patterns of relative group differences. In effect coding, the relative differences of codes are meaningful. A coding of 0 represents the grand mean of the observed data, whereas codings of either under or over 0 represent relative deviations from the grand mean. Positive effect codes mean that the condition that has been assigned the code is expected to score above the grand mean, whereas a negative code means that the condition is expected to score below the grand mean.

Since there were four different groups in this experiment, three contrasts needed to be tested to fully account for all degrees of freedom (see Table 3 for the focal and residual contrasts).

**Table 3:** **Contrast coding for Experiments 1, 2**.

**Goal condition**	**Experiment 1: interesting, task-irrelevant information/Experiment 2: pending goal**			
	**Without**		**With**	
**Task difficulty**	**Easy**	**Difficult**	**Easy**	**Difficult**
**PROBLEM-SOLVING PERFORMANCE**				
Focal contrast	+3	−1	−1	−1
Residual contrast 1	0	−1	2	−1
Residual contrast 2	0	1	0	−1
**TIME ON TASK−RELEVANT INFORMATION**				
Focal contrast	+1	+1	−3	+1
Residual contrast 1	−2	+1	0	+1
Residual contrast 2	0	−1	0	+1
**TIME ON TASK−IRRELEVANT INFORMATION**				
Focal contrast	−1	−1	+3	−1
Residual contrast 1	−1	−1	0	+2
Residual contrast 2	+1	−1	0	0

According to our main interaction hypothesis (Hypothesis 1), the presence of additional interesting information should lead to a reduction in problem-solving performance compared with the control condition when working on easy problems, but not when working on difficult problems. Accordingly, in the focal contrast the condition with easy test problems and no additional information was assumed to score best (coded +3), whereas the remaining three conditions were assumed to show worse performance (each coded −1)—either because of the higher difficulty of the problems or because the students were given the opportunity to retrieve interesting information while working on easy problems.

According to Hypothesis 2, participants in the condition with easy test problems and with additional interesting information should spend the least time on relevant information pages (coded −3), whereas more time on these pages should be spent in each of the remaining conditions (each coded +1). Moreover, these participants should also spend more time on task-irrelevant information. Since there was no irrelevant information in the control condition, this was tested by comparing the two conditions with interesting information regarding the time students spent on this information with a *t*-test. If students were more vulnerable to distraction when solving easy compared with more difficult problems, then they should also process more of the irrelevant information in the prior than in the latter case.

Finally, mediation analyses (Preacher and Hayes, 2008) were planned to test whether changes in information processing behavior can explain differences in problem-solving performance (Hypothesis 3). In this analysis the total effect that the presence of task-irrelevant information has on problem-solving performance can be separated into the indirect effect that is mediated by the changes in information processing behavior and the remaining direct effect that cannot be explained by the mediating processing variables. A significant indirect effect and a non-significant direct effect would indicate that changes in the time spent on processing task-relevant and/or task-irrelevant information can explain a reduction in problem-solving performance when working on easy problems while additional interesting information is present.

### Results and discussion

#### Results

Means and standard deviations are displayed in Table 4.

**Table 4:** **Means (and standard deviations) as a function of interesting, task-irrelevant information and task difficulty (Experiment 1)**.

	**Interesting, task-irrelevant information**			
	**Without**		**With**	
**Task difficulty**	**Easy**	**Difficult**	**Easy**	**Difficult**
Problem-solving performance in % correct	75.23 (14.98)	60.65 (20.46)	74.73 (21.16)	62.09 (20.77)
Time on task-relevant information in seconds	1310.25 (418.24)	1473.88 (411.45)	1366.18 (527.87)	1317.18 (303.09)
Time on task-irrelevant information in seconds	–	–	22.12 (45.73)	111.71 (150.56)

In a first step, problem-solving performance was analyzed by means of a regression analysis in which the focal contrast and the two residual contrasts described earlier were entered simultaneously as predictors. The overall regression model was marginally significant only, *R*^2^ = 0.11, *F*_(3, 62)_ = 2.70, MSE = 382.48, *p* = 0.055. The results for the single predictors revealed that neither the focal contrast nor the second residual contrast explained variance to a sufficient degree (focal contrast: β = 0.20, *p* = 0.10; second residual contrast: β = −0.03, *p* = 0.83); however, the first residual contrast did, β = 0.27, *p* = 0.03. This latter contrast reflects the main effect of task difficulty, that is, students working on more difficult problems solved fewer problems correctly than those working on easier problems.

Secondly, the time on relevant information pages was analyzed by means of a regression analysis in which the focal contrast and the two residual contrasts described earlier were entered simultaneously as predictors. Because this variable was not normally distributed, we used the logarithmized (ln) values for the analysis. The overall regression model was not significant, *F* < 1. Thus, there were no differences in the time spent processing relevant information across the four experimental conditions.

Thirdly, we had a closer look at the time on task-irrelevant information in the two conditions where this information had been available. In the condition with easy problems, 41.2% of the students had retrieved additional information at least once for at least 12 s and at most 185 s. In the condition with more difficult problems, 52.9% of the students had retrieved additional information at least once for at least 33 s and at most 607 s. A *t*-test revealed marginally significant differences between the two conditions, *t*_(17.652)_ = −1.79, *p* = 0.09, which became significant when analyzing only the (logarithmized) data for those students who had retrieved interesting information, *t*_(14)_ = −2.20, *p* = 0.045. Importantly, in contrast to our hypothesis, students working on more difficult problems tended to process task-irrelevant information for a longer time than students working on easier problems. However, it is important to bear in mind that these findings reliably hold only for less than half of the sample investigated.

Because there had been no evidence for negative effects of interesting, task-irrelevant information on either problem-solving performance or time on relevant information as well as differences in processing task-irrelevant information in contrast to our initial assumption, we refrained from running the mediation analyses described above.

#### Discussion

The results showed that adding interesting, but task-irrelevant information was suited to evoke students' interest—at least in some of them. In particular, students who had to solve difficult test problems were slightly more prone to take the bait and to retrieve and process the irrelevant information. This finding stands in contrast to what would have been predicted based on volitional action control theory. According to this theory more difficult tasks should serve to protect the main goal (i.e., learning and problem solving) from distractions that arise during task accomplishment, whereas students working on easier problems should be more vulnerable to engage in off-task activities.

It is important to note that students in the present experiment even though they processed task-irrelevant information showed no performance decrements. Thus, the opportunity to form transient browsing goals did not affect students' problem-solving performance nor did it lead to less processing of task-relevant information. To conclude, transient browsing goals even though they may lead to observable off-task behavior may not be strong enough to lead to visible aversive effects regarding performance. Thus, students were able to keep their task goal on track.

In Experiment 2 we investigated whether this pattern of results would change if the task-irrelevant information was related to a pending task that had to be accomplished subsequently to the learning and problem-solving task.

## Experiment 2

In this experiment we investigated whether pending goals that arise from tasks that have to be accomplished subsequently to completing the primary task compete with the latter goal for execution. Based on models of volitional action control we predicted that effects of goal competition would be observable and would be moderated by the primary task's difficulty (Heise et al., 1997; Czerwinski et al., 2000a,b). That is, we expected that problem-solving performance would be impaired due to pending goals for students working on easy problem-solving tasks, but not for those solving more difficult test problems. Moreover, performance impairments were assumed to be associated with and potentially caused by less time spent on processing task-relevant information and more time spent on processing task-irrelevant information, respectively.

### Materials and methods

#### Participants and design

Sixty-eight students (41 female, 27 male) of the University of Goettingen, Germany, participated in the experiment for either course credit or payment. Average age was 24.75 years (*SD* = 4.87). Participation was voluntary. All participants had taken a course in introductory statistics and were therefore familiar with the domain chosen for experimentation. The study was based on a between-subjects 2 × 2-design with task difficulty (easy vs. difficult) and presence of pending goal related to interesting, task-irrelevant information (with vs. without) as independent variables. Seventeen students were randomly assigned to each of the four experimental conditions.

#### Materials

The same hypermedia environment on combinatorics as in Experiment 1 was used for experimentation. HyperComb consisted of a short introduction to the domain and a learning and problem-solving phase, which comprised three test problems as well as one worked-out example for illustrating each of the six problem categories. As in Experiment 1, depending on experimental condition the test problems were either easy or more difficult to solve. The worked-out examples were embedded in interesting cover stories related to issues of attractiveness and mate choice. In all versions of the environment, hyperlinks were embedded in the example problem that allowed for retrieving additional information. The interestingness and the relevance of this information for the pending goal depended on experimental condition. In *conditions with pending goal* interesting, task-irrelevant information was linked to the worked-out examples, which was identical to the respective condition of Experiment 1. Additionally, to induce a pending goal participants were informed that they would have to work on a second task within the same hypermedia environment after having finished the problem-solving task. This second task consisted in answering three questions about attractiveness and mate choice that were presented at the beginning of the experiment (e.g., which eight factors are most important in influencing mate choice?). Participants were instructed to work on the problem-solving task first and to postpone thinking about the question-answering task until they had finished the word problems. They were assured that they would have enough time afterwards to browse the hypermedia environment for information relevant to this second task. Because the information on attractiveness and mate choice was of relevance to this explicit second task it provided an opportunity for participants to execute activities related to the pending question-answering task. In the condition *without pending goal* definitions of legal terms (e.g., proof, investigation, procedure) were linked to the worked examples. This information was supposed to be of no great interest to the learners. It was used since on the one hand we wanted to avoid the formation of transient browsing goals, while on the other hand ensuring that the hypermedia environment contained the same amount of task-irrelevant information as in the pending-goal condition. Participants were instructed to work on the learning and problem-solving task and no second task was announced to them.

#### Dependent variables

Students' problem-solving performance, the time spent processing relevant information pages as well as the time spent on irrelevant information pages were assessed in the same manner as in Experiment 1.

#### Procedure

The procedure was identical to that of Experiment 1 with one exception. In the conditions with pending goal, students were told that they would have to work on a second task after having finished the primary task before they started working on the test problems.

#### Data analyses

The data were analyzed using regression analyses along with effect coding and mediation analyses analogously to Experiment 1. The focal and residual contrasts for the regression analyses are shown in Table 3.

### Results and discussion

#### Results

Means and standard deviations are displayed in Table 5.

**Table 5:** **Means (and standard deviations) as a function of a pending goal related to interesting, task-irrelevant information and task difficulty (Experiment 2)**.

**Task difficulty**	**Pending goal**			
	**Without**		**With**	
	**Easy**	**Difficult**	**Easy**	**Difficult**
Problem-solving performance in % correct	77.78 (11.03)	61.66 (19.64)	60.78 (21.87)	59.91 (20.67)
Time on task-relevant information in seconds	1469.12 (633.70)	1226.06 (379.85)	950.24 (429.49)	1211.76 (916.13)
Time on task-irrelevant information in seconds	2.18 (4.59)	7.06 (14.35)	105.29 (228.76)	34.47 (71.80)

In a first step, problem-solving performance was analyzed by means of a regression analysis in which the focal contrast and the two residual contrasts were entered simultaneously as predictors. The overall regression model was significant, *R*^2^ = 0.14, *F*_(3, 64)_ = 3.50, MSE = 353.32, *p* = 0.02. The results for the single predictors revealed that only the focal contrast was a significant predictor, β = 0.37, *p* = 0.002 (first residual contrast: β < 0.01, *p* > 0.99; second residual contrast: β = 0.03, *p* = 0.79). Accordingly, as expected, students working on easy problems without pending goal achieved the best problem-solving performance. Students working on easy problems with pending goal, however, showed a similar performance as students working on more difficult problems.

Secondly, the time spent on relevant information pages was analyzed. Because this variable was not normally distributed, we used the logarithmized (ln) values for the analysis. The overall regression model was significant, *R*^2^ = 0.13, *F*_(3, 64)_ = 3.25, MSE = 0.20, *p* = 0.03. This effect could be traced back completely to the variance being explained by the focal contrast, β = 0.31, *p* = 0.01 (first residual contrast: β = −0.17, *p* = 0.15; second residual contrast: β = 0.09, *p* = 0.46). Accordingly, students working on easy problems when a pending goal was present spent less time processing task-relevant information pages compared with the remaining conditions.

Thirdly, we analyzed the time on task-irrelevant information. There were only relatively few students in each condition, who had actively retrieved additional irrelevant information, whereby those who did differed largely in the time spent processing this information: with pending goal—easy problems: 29.4% of students, min. duration: 57 s, max. duration: 803 s; with pending goal—difficult problems: 29.4% of students, min. duration: 14 s, max. duration: 248 s; without pending goal—easy problems: 29.4% of students, min. duration: 4 s, max. duration: 18 s; without pending goal—difficult problems: 41.2% of students, min. duration: 4 s, max. duration: 53 s). Despite differences in the variance among conditions, we analyzed the time on irrelevant information by means of a regression assuming that this analysis is sufficiently robust against violations of homogeneity assumptions. The regression revealed a marginally significant overall model, *R*^2^ = 0.33, *F*_(3, 64)_ = 2.66, MSE = 1442.52, *p* = 0.055. This effect could be traced back completely to the variance being explained by the focal contrast, β = 0.32, *p* = 0.009 (first residual contrast: β = 0.10, *p* = 0.41; second residual contrast: β = −0.01, *p* = 0.91). Accordingly, students working on easy problems when a pending goal was present spent more time processing task-irrelevant information pages compared with the remaining conditions. However, it is important to note that this effect was driven by a small subgroup of students, whereas most participants did not retrieve any of the task-irrelevant information at all.

To summarize, in line with predictions derived from volitional action control theory students solving easy problems when a pending goal related to interesting information was present solved less problems correctly, processed task-relevant information for a shorter time and task-irrelevant information for a longer time compared with the remaining conditions. This raises the issue whether the latter changes in overt information processing behavior can be used to explain the negative effects found for problem-solving performance. To answer this question, we had a closer look at only the two conditions with easy test problems and ran two mediation analyses. Condition (with vs. without pending goal) served as predictor, time on task-relevant information and on task-irrelevant information served as mediators, respectively, and problem-solving performance was the dependent variable. In both mediation analyses the mediators did not allow explaining differences between conditions in problem-solving performance, that is, there were no significant indirect effects (with time on task-relevant information as mediator: *z* = 1.38, *p* = 0.17; with time on task-irrelevant information as mediator: *z* = −1.24, *p* = 0.22). Thus, even though differences in information processing behavior accompanied the effects of a pending goal on problem-solving performance, the prior was unsuited to explain the latter.

The finding that changes in performance occurred independently of processing task-irrelevant information was corroborated by additional exploratory analyses that were conducted only with students, who had never clicked on the task-irrelevant information (*N* = 46). If performance differences among conditions were caused by processing task-irrelevant information, then we should be unable to confirm our hypotheses for these students. However, rerunning the regression analyses for problem-solving performance and for time on relevant information with only these students revealed the same pattern of results as when analyzing the data of all students. That is, also those students who had never exerted any overt distraction behavior showed reduced performance (overall regression model: *R*^2^ = 0.20, *F*_(3, 42)_ = 3.56, MSE = 360.09, *p* = 0.02; focal contrast: β = 0.44, *p* = 0.003) and limited processing of task-relevant information (overall regression model: *R*^2^ = 0.18, *F*_(3, 42)_ = 2.80, MSE = 0.24, *p* = 0.051; focal contrast: β = 0.38, *p* = 0.009) when a pending goal was present and they had to solve easy problems. All residual contrasts were non-significant (all *ps* > 0.30).

#### Discussion

The results from Experiment 2 corroborate our hypotheses to a large extent. In line with Hypothesis 1, a pending goal related to interesting, task-irrelevant information reduced problem-solving performance for students working on easy problems, but not for those working on more difficult ones. Thus, the results confirm predictions derived from volitional action control theories suggesting that a difficult task helps to protect the primary goal from interference caused by a pending goal (e.g., Heise et al., 1994a,b). Moreover, confirming Hypothesis 2, this was accompanied with changes in students' information processing behavior in that students working on easy problems in the pending goal condition processed task-relevant information for a shorter time and task-irrelevant information for a longer time compared with students in the remaining conditions. At first sight these results seem to align with the assumption of Harp and Mayer (1998) as well as Lehman et al. (2007) that the withdrawal of attention away from the relevant toward the irrelevant information is a cause for negative performance effects found in the context of seductive details research. However, in contrast to Hypothesis 3, in the present study these changes in information processing behavior were not causally related to performance; rather, they appeared to be a mere by-product of it. Moreover, even students who did not show any overt distraction behavior were negatively impacted by the presence of a pending goal when working on easy problems. Possible alternative explanations will be discussed in the following section.

## General discussion

Our experiments were designed to explore the effects of goal competition on task performance and information processing in hypermedia-based learning and problem solving. In line with theories of volitional action control we were able to demonstrate impairments in problem-solving performance when task-irrelevant information was embedded within a hypermedia environment that was related to an explicit pending goal that users were instructed to pursue later. As had been expected in Hypothesis 1, these performance impairments were observable only for students working on easy problems, but not for those working on more difficult problems, thereby confirming prior findings by Heise et al. (1994b, 1997) using a more complex setting. Importantly, performance impairments were not triggered by the mere availability of interesting task-irrelevant information as could be demonstrated in Experiment 1 and as has been furthermore shown by Heise et al. (1994b). Thus, it seems that at least in an experimental laboratory context transient browsing goals are not sufficiently strong to interfere with a learning and problem-solving task that students have been instructed to work on, which is line with findings by Barab et al. (1996). This also corresponds with findings from Harp and Mayer (1998); Harp and Mayer (Experiment 2), who found that introducing learning objectives to support the main (learning) goal led to a significant reduction of the seductive details effect. Goals appear to be useful to constrain users' information search so that users are prevented from getting distracted by task-irrelevant information—at least as long as this information is not related to a pending goal of the user.

Experiment 2 also provided evidence in favor of Hypothesis 2 in that the presence of a pending goal led to more time being spent on the processing of the task-irrelevant information as well as to less time being spent on the processing of the task-relevant information—at least when working on easy problems. There were no comparable effects in Experiment 1. At first sight the changes in information processing behavior appear to be a likely cause of the performance impairments found in Experiment 1 (cf. Harp and Mayer, 1998; Lehman et al., 2007). That is, if students reallocate cognitive resources toward processing task-irrelevant information at the expense of task-relevant information, this is likely to yield worse performance in the problem-solving task (cf. Naumann et al., 2007). However, there was no evidence in our data to confirm this plausible assumption that was addressed in Hypothesis 3. The time spent processing relevant information proved to be no significant mediator for the negative effect of a pending goal on problem-solving performance. Likewise, overt distraction behavior in terms of processing task-irrelevant information did not explain performance impairments. First, it did not mediate the negative effect of a pending goal on problem-solving performance. Second, performance impairments were also observable for students not showing any overt distraction behavior. Moreover, the latter students were in the majority. Third, comparing the conditions from Experiments 1 and 2 that had available interesting, task-irrelevant information (Experiment 1: transient goal conditions; Experiment 1: pending goal conditions) shows that even though in both experiments at least some students processed the interesting information, performance impairments were only observable in Experiment 2. Thus, overt changes in information processing behavior are an unlikely cause of the negative impact of pending goals on task performance. Accordingly, difficult tasks appear to help keep task goals on track in the sense that students are able to maintain a reasonable level of performance even when on a behavioral level they show over distraction behavior.

The hypothesis that overt changes in information processing behavior cause performance impairments had been derived from research on the seductive details effects, where overt distraction behavior (at the expense of processing task-relevant information) is discussed as one possible explanation for why seductive details hamper learning (Harp and Mayer, 1998; Lehman et al., 2007; Rey, 2012). Importantly, even though Lehman et al. (2007) found result patterns that seem to be in line with the idea that changes in overt information processing behavior cause performance impairments, the causal link between these two aspects has not been tested.

The question arises what then causes performance impairments, if not overt distraction behavior. In the literature on the seductive details effect, diversion, disruption, and depletion are discussed as alternative explanations (Harp and Mayer, 1998; Rey, 2012). In our case, it seems unlikely that the interesting information triggered inappropriate schemas for encoding the task-relevant information (diversion) or that its processing interrupts the construction of a coherent mental representation of the relevant information (disruption). In both cases, one would have expected negative effects on problem-solving performance whenever the task-irrelevant information was processed, which was not the case in the reported experiments. The explanation that appears to best match our data is based on a cognitive-resources account (depletion).

According to the depletion explanation, performance impairments arise if task-irrelevant information demands cognitive resources, which are then no longer available for the main task. Theories of volitional action control suggest that it is not the interesting information *per se* that causes negative effects, but the fact that it is related to a pending goal, which is well in line with the present findings. Pending goals reside in memory with a heightened state of activation (Goschke and Kuhl, 1993; Marsh et al., 1999); thus, they withdraw (attentional) resources from the main task (cf. Li et al., 2000). Also deliberating on whether to follow the current or the pending goal as well as suppressing action tendencies to follow the pending goal is likely to claim resources (cf. ego depletion effect, Baumeister et al., 2000). Similarly, Wegner et al. (1987) have suggested that suppressing a thought (e.g., related to a pending goal) may require cognitive resources and be time-consuming. Most importantly, suppressed thoughts may easily return to consciousness when triggers appear in the environment. In our case, hyperlinks providing access to information that was relevant for the pending goal may thus have activated thoughts regarding the pending goal whenever they were encountered and these thoughts interfered with working on the main task. Combining these different strands of research thus allows specifying the depletion explanation: Task-irrelevant information leads to performance impairments if it is linked to a pending goal, in which case there will be a goal conflict in memory, where the current goal and the pending goal compete for limited cognitive resources. Importantly, this requires cognitive resources without necessarily leading to observable engagement with the pending goal, explaining why even students, who do not show overt distraction behavior, suffer from performance impairments.

Such an account would also fit nicely with findings from Sanchez and Wiley (2006), who found a seductive details effect only for students performing low in a working memory task that measured their ability to control attention and to stay focused on a specific goal (Kane et al., 2001). These students should be especially vulnerable to forming transient browsing goals even in the presence of an instructed primary goal and should easily suffer from goal conflicts, explaining why they showed a strong seductive details effect. Moreover, the idea that goal conflicts demand cognitive resources is well in line with hypermedia research suggesting that decisions on whether to retrieve a specific information, which may either be related to the current or the pending goal (i.e., navigational decisions), are one potential source of cognitive overload (Niederhauser et al., 2000). Cognitive overload is seen as one reason for why learning with hypermedia is often not more or even less effective than linear, system-controlled instruction despite its envisioned advantages (Scheiter and Gerjets, 2007; Scheiter, 2014). These problems should become even more evident when considering that many students have difficulties in deciding whether or not a piece of information is relevant to the task at hand once the information space becomes larger and potentially more ambiguous (e.g., in the Internet, Braasch et al., 2009; Goldman et al., 2012).

Even though the results reported in this paper are well in line with previous research, the results require further replication since the present studies were based on relatively small sample sizes. Moreover, because students were allowed to decide whether or not to retrieve the task-irrelevant information, there was huge variability regarding this aspect. Thus, any of the quantitative analyses regarding differences among conditions in students' overt distraction behavior need to be handled with care since only a minority of students actually showed distraction behavior. Importantly, in the present case even though this makes any statistical claims difficult it allowed us to derive important insights, namely, that overt distraction behavior is an unlikely cause of performance impairments.

There are various avenues for future research. First, in the present paper no impact of transient search goals could be observed, which could be due to the experimental situation. Distraction effects due to the activation of personal interests are probably restricted to more natural situations (e.g., browsing the Internet) or to situations where the task-irrelevant information cannot be avoided (cf. seductive details research). Thus, it should be investigated whether transient search goals emerge in more natural Internet browsing scenarios and if so, whether their effects are moderated by the difficulty of the search task, as would be predicted by theories of volitional action control. Second, future research should aim at finding process indicators for the depletion account earlier. For instance, eye tracking could be used to study whether students deliberate for a longer time whether or not to click a link leading to task-irrelevant information when working on easy tasks in the presence of a competing goal. Deliberation should be evident when students attend for a longer time to the respective hyperlinks and move their eyes more frequently between task-relevant and task-irrelevant links (cf. Gerjets et al., 2011, for an application of the eye-tracking method when studying information search). Third, studying the role of individual differences in the paradigm used in the studies reported in this paper could be one way of finding further evidence for the depletion explanation. If goal conflicts are dependent on a person's resources to control their attention, then these resources should moderate the effects of pending goals (cf. Sanchez and Wiley, 2006). This might also imply that effects are different for younger children, whose ability to control attention is still under development or for people with attention control deficits such as children with Attention Deficit and Hyperactivity disorders (cf. Gerjets et al., 2002). Similarly, a person's action orientation, that is, a person's propensity to act and to pursue goals (cf. Kuhl, 1984), should influence how well students are able to accomplish a current task in the face of goal competition (Corno and Kanfer, 1993). Finally, it is important to note that in the present studies task difficulty was manipulated experimentally as a between-subjects variable by choosing different tasks. However, task difficulty will also vary depending on a student's prior knowledge. Thus, it would be interesting to study how students with varying levels of prior knowledge would respond to the presence of a pending goal. On the one hand, one could argue that students with higher compared with lower levels of prior knowledge should be more prone to distraction, since for the prior a given problem is easier than for the latter. On the other hand, once students have prior knowledge available, they can activate more task-relevant concepts in memory, which may possible make them less susceptible to influences of a conflicting goal.

Importantly, the present research has been carried out under the assumption that getting distracted is something negative, because it endangers the accomplishment of a specific educational goal (e.g., finding solutions to clearly defined test problems, acquisition of cognitive skills, memorization of facts). However, providing vast amounts of information may nevertheless be useful for incidental learning. These positive effects of providing a wide range of heterogeneous information for exploratory learning are sometimes described as “serendipity effects” (Kuhlen, 1991). Future studies that use a learning environment with vast amounts of information in combination with varying learning goals may shed light on these possible positive effects of providing potentially distracting information.

### Conflict of interest statement

The authors declare that the research was conducted in the absence of any commercial or financial relationships that could be construed as a potential conflict of interest.
